# Structural Brain Connectivity Constrains within-a-Day Variability of Direct Functional Connectivity

**DOI:** 10.3389/fnhum.2017.00408

**Published:** 2017-08-10

**Authors:** Bumhee Park, Jinseok Eo, Hae-Jeong Park

**Affiliations:** ^1^Department of Statistics, Hankuk University of Foreign Studies Yong-In, South Korea; ^2^BK21 PLUS Project for Medical Science Seoul, South Korea; ^3^Department of Nuclear Medicine, Radiology, and Psychiatry, Severance Hospital, Yonsei University College of Medicine Seoul, South Korea; ^4^Department of Cognitive Science, Yonsei University Seoul, South Korea; ^5^Center for Systems and Translational Brain Sciences, Institute of Human Complexity and Systems Science, Yonsei University Seoul, South Korea

**Keywords:** functional magnetic resonance imaging, resting state functional connectivity, partial correlation matrix, within-a-day variability, functional connectivity dynamics

## Abstract

The idea that structural white matter connectivity constrains functional connectivity (interactions among brain regions) has widely been explored in studies of brain networks; studies have mostly focused on the “average” strength of functional connectivity. The question of how structural connectivity constrains the “variability” of functional connectivity remains unresolved. In this study, we investigated the variability of resting state functional connectivity that was acquired every 3 h within a single day from 12 participants (eight time sessions within a 24-h period, 165 scans per session). Three different types of functional connectivity (functional connectivity based on Pearson correlation, direct functional connectivity based on partial correlation, and the pseudo functional connectivity produced by their difference) were estimated from resting state functional magnetic resonance imaging data along with structural connectivity defined using fiber tractography of diffusion tensor imaging. Those types of functional connectivity were evaluated with regard to properties of structural connectivity (fiber streamline counts and lengths) and types of structural connectivity such as intra-/inter-hemispheric edges and topological edge types in the rich club organization. We observed that the structural connectivity constrained the variability of direct functional connectivity more than pseudo-functional connectivity and that the constraints depended strongly on structural connectivity types. The structural constraints were greater for intra-hemispheric and heterologous inter-hemispheric edges than homologous inter-hemispheric edges, and feeder and local edges than rich club edges in the rich club architecture. While each edge was highly variable, the multivariate patterns of edge involvement, especially the direct functional connectivity patterns among the rich club brain regions, showed low variability over time. This study suggests that structural connectivity not only constrains the strength of functional connectivity, but also the within-a-day variability of functional connectivity and connectivity patterns, particularly the direct functional connectivity among brain regions.

## Introduction

One of the important questions about the brain is the emergence of dynamic functionalities from a stable structure. A growing number of studies have recently been conducted to explore this structure-function relationship in terms of the large-scale brain network, composed of nodes (interaction units) and their interactions, called edges (Honey et al., 2009; van den Heuvel et al., 2009; Hermundstad et al., 2013; Goni et al., 2014). Most of these studies are facilitated by two non-invasive neuroimaging techniques, resting state functional magnetic resonance imaging (rs-fMRI) for functional networks (Biswal et al., 1995; Greicius et al., 2003; Raichle and Snyder, 2007) and diffusion tensor imaging (DTI) (Basser et al., 1994) for structural networks (see Park and Friston, 2013 for a review).

In studies with those imaging techniques, functional networks are strongly coupled to or constrained by structural networks. For example, Honey et al. (2009) and Hermundstad et al. (2013) have shown strong positive correlations between structural connectivity (fiber streamline counts of DTI tractography) and functional connectivity (cross-correlations among regional fMRI signals). Most of the studies that explored structural constraints on the functional brain networks (Honey et al., 2009; van den Heuvel et al., 2009; Hermundstad et al., 2013; Goni et al., 2014) were based on the assumption of stable functional over the time. However, recent studies have shown the dynamic nature of functional connectivity, even during a single session of rs-fMRI acquisition (Chang and Glover, 2010; Cribben et al., 2012; Handwerker et al., 2012; Hutchison et al., 2013; Kucyi et al., 2013; Allen et al., 2014; Calhoun et al., 2014; Monti et al., 2014; Zalesky et al., 2014), which raises new questions. Do large fiber bundles interconnecting two brain regions mediate temporal variability in functional connectivity, or reduce the variability of functional connectivity between the two regions? Do the structural constraints on functional connectivity differ across edge types? These questions of how the structural connectivity is associated with the “variability” or dynamicity of functional connectivity remain unresolved.

Several studies have explored structural constraints on the variability of functional connectivity (Liao et al., 2015; Liegeois et al., 2016; Zhang et al., 2016). Liao et al. (2015) reported that homologous inter-hemispheric functional connections have lower temporal variability than heterologous inter-hemispheric connections. Intra-modular edges showed lower variability of functional connectivity than inter-modular edges (Zhang et al., 2016). Liegeois et al. (2016) showed increased similarity of the structural network to less efficient (in message passing in the graph theory) and to more highly modular functional network states during periodic functional network fluctuation. These studies have explored short-time range (micro-state) variability of conventional (Pearson-correlation based) functional connectivity over the structural connectivity within a single session of 10 min.

In the current study, using rs-fMRI data acquired every 3 h within a day (Park et al., 2012), we investigated the structural constraints on the variability of temporal meso-scale functional connectivity in the three following aspects.

**Figure 1:**
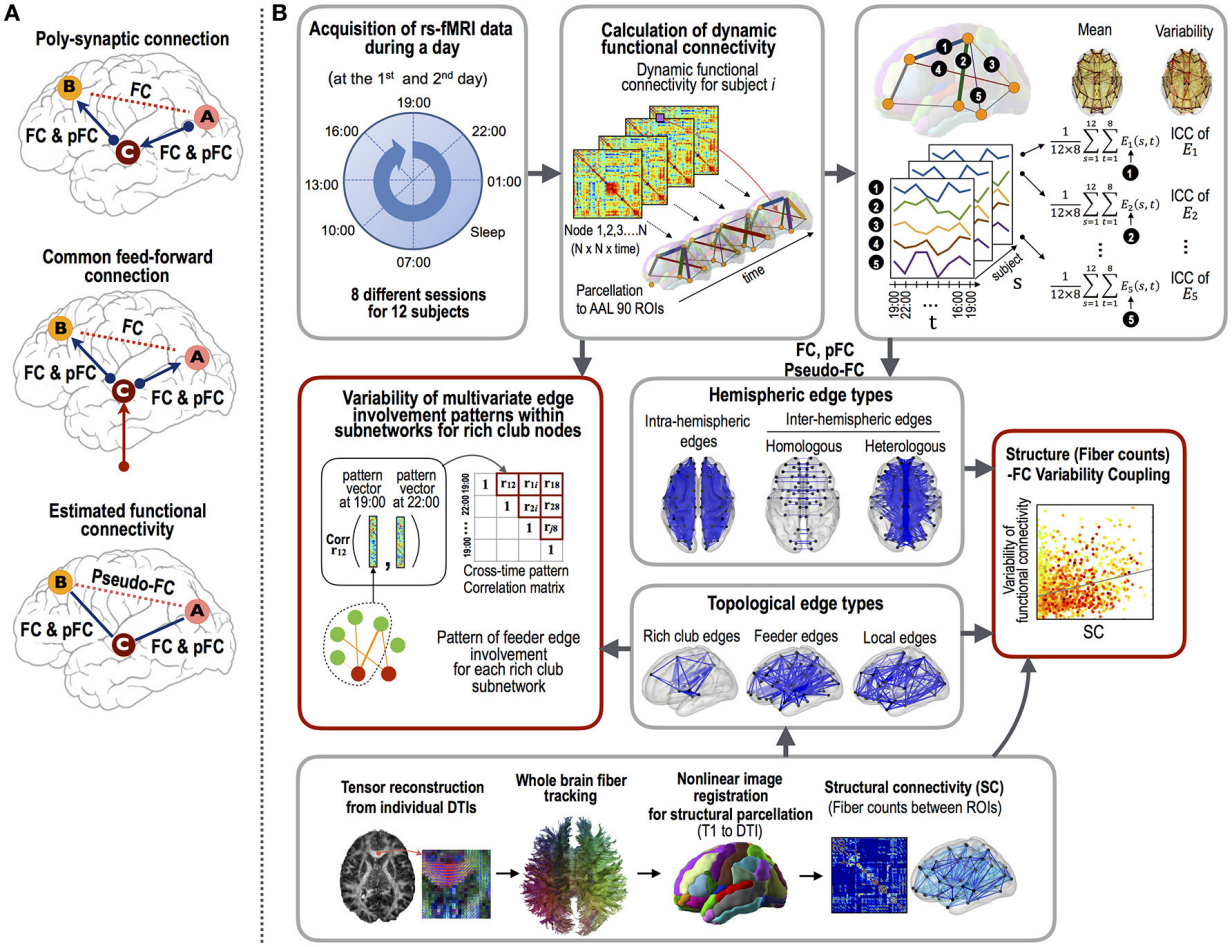
The procedures employed for the functional connectivity variability analysis.**(A)** The three types of functional connectivity: FC, pFC, and pseudo-FC. Functional connectivity was defined by Pearson correlation coefficient (FC) and partial correlation (pFC). Pseudo-functional connectivity (pseudo-FC) was defined as the difference between FC and pFC, i.e., the edges where FC exists but pFC does not. Pseudo-FC can be derived with polysynaptic connections or common feed-forward projections without direct interactions between two regions. **(B)** The analyses used in this study were as follows: (1) the acquisition of resting state fMRI data at eight different time points during a day and DTI data, (2) calculation of three types of functional connectivity (i.e., FC, pFC, and pseudo-FC) and structural connectivity (i.e., log-transformed fiber counts), (3) correlation analyses between intra-class correlations (ICCs, stability) of the functional connectivity and structural connectivity according to edge properties (fiber counts and lengths), edge types (intra-hemispheric and inter-hemispheric homologous and heterologous edges), and topological edge types under rich club architecture, and (4) evaluations of the multivariate edge involvement pattern similarity under the rich club architecture. The multivariate pattern similarity was evaluated by calculating the average similarities of the connectivity matrices within subnetworks of the rich club nodes across the different time points within a day. Note that variability was evaluated with similarity measures (1–similarity).

First, we explored the variability of three different types of functional connectivity measures. Currently, Pearson cross-correlation coefficients across an fMRI time series are conventionally used as a gauge of the functional connectivity between two brain regions. However, this measure cannot factor out any latent effects of a third and/or other nodes that simultaneously modulate the paired nodal activities (Gerstein and Perkel, 1969) (Figure 1A). This makes the interpretation of the functional connectivity, whether they are from the direct connections *per se* or from an indirect polysynaptic induction or modulatory effects, unclear. In order to evaluate direct interactions (or connectivity), researchers have utilized a partial correlation analyses of fMRI time series (Marrelec et al., 2006; Smith et al., 2011). As explained in Figure 1A, the partial correlation-based functional connectivity (pFC) may not exist at edges where Pearson correlation-based functional connectivity (FC) exists. We call the differences between FC and pFC as pseudo-functional edges (pseudo-FC), which are edges where FC exists but pFC does not. These three types of functional connectivity (FC, pFC, and pseudo-FC) reflect different aspects of functional interactions and may reveal distinctive variability over the structural white matter connectivity (SC).

Second, we associated the variability of functional connectivity and structural connectivity properties according to structural edge types. Particularly, we subdivided structural edges into the intra-hemispheric edges, which connect regions within each hemisphere, homologous inter-hemispheric edges, which connect inter-hemispheric homologous regions, and heterologous inter-hemispheric edges. This subdivision has previously been used to explore structural constraints on within-session variability of functional connectivity (Liao et al., 2015; Shen et al., 2015b). Studies have also implicated topological edge (type)-specific variability of functional connectivity in the resting brain. For instance, Zalesky et al. (2014) reported that temporal variations in functional network properties occur mostly in inter-modular edges. Thus, we also explored the contribution of the topological properties of structural edges to the variation in functional connectivity. More specifically, we differentiated structural edges according to a rich club architecture (van den Heuvel and Sporns, 2011), which has been used to explain brain function in the respect of global integration of segregated brain regions (van den Heuvel and Sporns, 2013; Collin et al., 2014; Jang et al., 2017; Liang et al., 2017). Rich club organization includes highly interconnected rich club nodes as hubs, feeders (edges) connecting with rich clubs, and locals (edges) connecting non-rich club nodes and feeder nodes (van den Heuvel and Sporns, 2011). In this architecture, rich club hubs play not only centers of local segregation but centers for global integration. In this respect, we considered that the rich club architecture may well be association with variable functional connectivity compared to community structures defined by modularity optimization (Newman, 2006), which primarily focus on functional segregation.

Third, we evaluated the variability of multivariate edge involvement patterns, which are a set of functional interactions, in subnetworks. A single functional edge in a network does not make a brain function by itself, but it needs to participate in a subnetwork, which is a multitude of edges that temporarily congregate together in a certain context (McIntosh, 2000; Shanahan, 2012). This hypothesis is supported by the discovery of a pool of network subcomponents that are embedded in the resting brain (Park et al., 2014). Accordingly, we explored the within-a-day variations of functional interaction patterns in different types of subnetworks, including subnetworks of hubs (i.e., rich clubs), feeders, and locals in the rich club organization.

## Materials and methods

### Data and image processing

In this study, we reanalyzed the data set reported in Park et al. (2012). Briefly, data from 12 healthy, right-handed participants (9 males and 3 females, mean age 25.42 ± 2.84 years) were used in this evaluation. Each subject was scanned using resting-state fMRI protocol for 5.5 min at eight different times of the day: 19:00 (1st day), 21:00, 1:00 (2nd day), 7:00, 10:00, 13:00, 16:00, and 19:00. All patients provided written informed consent before procedures and this study received Institutional Review Board of Yonsei University Severance Hospital.

Resting-state fMRI data were acquired axially using T2^*^ weighted single shot echo planar imaging (EPI) sequences a 3.0 Tesla MRI scanner (Siemens Tim Trio, Erlangen, Germany): voxel size, 3.0 × 3.0 × 3.3 mm^3^; slice number, 32 (interleaved); matrix, 64 × 64; slice thickness, 3.3 mm; repetition time (TR), 2,000 ms; echo time (TE), 30 ms; and field of view, 192 mm. Each 330-s scan produced 165 fMRI images. During each resting-state fMRI scanning session, subjects were instructed to keep their eyes closed, without falling asleep or thinking about something specific. After the scanning, subjects were asked to report their overall physical condition including sleepiness.

All participants stayed freely within the institute with a routine light exposure during the scanning day with an instruction of abstaining from highly demanding physical or mental works, alcohols, or nicotine. Each participant was emphasized not to sleep during each scan and no participants slept according to self-reports after scanning. Since this study is aimed to investigate within-a-day variability of routine functional networks rather than the circadian rhythm, we did not tightly control factors relating to circadian oscillations or time-of-day effects.

A high-resolution structural data set was also taken from each subject using a magnetization-prepared rapid acquisition gradient echo (MP-RAGE) three-dimensional T1-weighted sequence (voxel size, 0.9 × 0.9 × 1.0 mm^3^; TR, 2,300 ms; TE, 3.08 ms). Diffusion tensor images were obtained using single-shot echo-planar acquisition from 45 non-collinear, non-coplanar diffusion encoded gradient directions with the following parameters: 128 × 128 acquisition matrix with 70 slices, 220-mm field of view, 1.72 × 1.72 × 2 mm^3^ voxels, TE 60 ms, TR 7.384 s, b-factor of 600 s/mm^2^, without cardiac gating. Foam pads were used to reduce head motion during all MRI data acquisition.

fMRI data preprocessing was conducted using statistical parametric mapping (SPM12, http://www.fil.ion.ucl.ac.uk/spm/, Wellcome Trust Centre for Neuroimaging, London, UK) (Friston et al., 1994). After discarding the first 5 scans due to some stability issues, all EPI data were preprocessed by correcting for the delay in the acquisition time between different slices, and correcting for head motion by realignment of all consecutive volumes to the first image of the session. The realigned images were co-registered to T1-weighted images, which were used to spatially normalize functional data into a template space using non-linear transformation. We did not conduct spatial smoothing on the fMRI data to avoid inflation of local connectivity and clustering.

### Functional network construction

Figure 1B summarizes all the evaluation processes conducted in this study. We extracted fMRI time series from the 90 regions of the AAL map. FMRI time courses were processed through (1) regressing out effects of six rigid motions and their derivatives, and three principal components the white matter and the cerebrospinal fluid mask segmented using SPM12; (2) spike detection and despiking based on four times of the median absolute deviation; and (3) band-pass filtering (0.009–0.08 Hz) (Weissenbacher et al., 2009; Power et al., 2012; Taylor et al., 2014; Thomas et al., 2014).

All procedures were performed using in-house multimodal brain network analysis software, MNET (multimodal brain network analysis toolbox; Yonsei University, http://neuroimage.yonsei.ac.kr/mnet). We defined individual functional networks using two different methods; (1) Pearson correlation matrix (i.e., FC) and (2) regularized estimation of partial correlation matrix (i.e., pFC) among 90 regional mean fMRI time-series.

A graphical LASSO (least absolute shrinkage and selection operator) method was used to estimate pFC (Huang et al., 2010). Graphical LASSO aims to estimate a sparse matrix Θ (i.e., pFC matrix), maximizing the penalized Gaussian log-likelihood function as below:

$$\begin{array}{l} \text{L}\left(\Theta \right)-\lambda ||\Theta |{|}_{1}=log||\Theta ||-tr\left(\Theta \text{S}\right)-\lambda ||\Theta |{|}_{1} \end{array}$$

where ||·||, tr(·), and ||·||_1_ each denotes the determinant, trace, and *L*_1_ norm operator of the matrix, **S** is the sample covariance matrix, and λ is a regularization parameter controlling the level of sparsity for estimate of T. To determine optimal regularization parameter, λ, we applied stability approach to regularization selection (StARS) (Liu et al., 2010). For each session, StARS compared stabilities for λ candidates from 0.01 to 0.5 by increasing 0.01. FC and pFC were Fisher's r-to-z transformed before variability analyses.

As explained in the Introduction, we defined “pseudo functional edges” as a set of edges where FC exists but pFC does not and “direct functional edges” as edges where both FC and pFC exist. The threshold of the Pearson correlation coefficients for FC was set to *P* < 0.05 (Bonferroni corrected) for each subject in each session. PFC does not require any threshold since graphical LASSO drives the weak connectivity to zero during the estimation process. Pseudo-FC indicates FC over the pseudo functional edges.

### Structural network construction

We constructed structural and functional networks based on nodes defined by the 90 cerebral regions of the automated anatomical labeling (AAL) map (Tzourio-Mazoyer et al., 2002). Despite its scale effect on network properties (Zalesky et al., 2010) and regional inhomogeneity issues in functional networks (Park et al., 2013; Gordon et al., 2016), use of AAL map would make this study comparable with many previous studies.

To construct a structural network, we followed the approach that combined structural parcellation and whole brain white matter tractography (Park et al., 2004). We conducted automated fiber tracking of the diffusion tensor images using DoDTI (Yonsei University, http://neuroimage.yonsei.ac.kr/dodti), with the fourth order Runge-Kutta method and constructed whole white matter fiber bundles at ~300,000 white matter seed points. The stopping criteria for fiber tracking included a low fractional anisotropy (<0.2) and a rapid change of direction (>60 degree per 1 mm).

After registering AAL map and fiber tractography using linear affine transformation, fiber bundles crossing the AAL labels were extracted. Structural connectivity (SC) was defined as a fiber count between two brain regions on the AAL atlas, similarly to previous studies (Honey et al., 2009; van den Heuvel and Sporns, 2011; Hermundstad et al., 2013). A fiber length between a pair of two regions was defined by an average length of all fibers that interconnect the two regions.

After empirical evaluation of the distribution of fiber counts, we log-transformed fiber counts to improve the normality of the fiber count distribution, which is required for correlation analysis, more specifically, correlation analysis with functional connectivity measures in this study.

### Edge types in the structural network

We evaluated functional connectivity in terms of classifications based on two criteria—(1) *the location of connections:* intra-hemispheric, homologous inter-hemispheric and heterologous inter-hemispheric edges, and (2) *the topological role:* rich club, feeder, and local edges in light of the rich club architecture. Homologous inter-hemispheric edges refer to inter-hemispheric edges that connect homologous (corresponding) brain regions in the contra-lateral hemispheres while heterologous inter-hemispheric edges refer to the ones that connect heterologous brain regions across hemispheres.

Rich club edges were defined in the following steps. A network is called a rich club organization if the core nodes in the network are more strongly interconnected than expected by chance with a high degree of k. Such nodes are referred to as rich club nodes (van den Heuvel and Sporns, 2011). Rich club coefficient is used to determine whether the network has rich club organization or not by comparing the values of the network in question and the values of a randomly selected network (van den Heuvel and Sporns, 2011). Over a range of degree threshold, k, rich club coefficient, ϕ, is defined as follows:

$$\begin{array}{l} \phi \left(k\right)=\frac{{2E}_{>k}}{N_{>k}\left(N_{>k}-1\right)} \end{array}$$

where rich club coefficient, ϕ(*k*), is the ratio of actual number of edges between remaining nodes, *E*_>*k*_, and the total number of possible edges, *N*_>*k*_(*N*_>*k*_ − 1), between them, after removing all nodes with a degree less than *k*. Since the point of detecting rich clubs is to “categorize” each edge according to its topological role with other regions, rather than its fiber count levels, we calculated rich club coefficient in the binarized structural network as used in some previous studies (van den Heuvel and Sporns, 2011; Ball et al., 2014). Normalized rich club coefficient, ϕ_*norm*_, was calculated as ϕ_*norm*_(*k*) = ϕ(*k*)/ϕ_*rand*_(*k*), where ϕ_*rand*_(*k*) is the average value of ϕ(*k*) across 1,000 degree-preserving randomly generated networks. We performed one sample *t*-test with null hypothesis of ϕ_*norm*_(*k*) = 1 for each k and applied Bonferroni correction in the testing to control multiple testing across all examined levels of k. The presence of rich club organization is then determined if ϕ_*norm*_(*k*) > 1 for any range of k (*P* < 0.05, Bonferroni corrected). Nodes with a significant k degree on group-averaging structural connectivity (satisfying that fibers present for 50% subjects at least) were appointed as rich club nodes. After determining the rich club nodes, we categorized structural edges into three types: (1) edges between rich clubs (rich club edges), (2) edges between a rich club and a non-rich club (feeder edges), and (3) edges between non-rich clubs (local edges) (van den Heuvel and Sporns, 2011). See **Figure 5B**.

### Within-a-day variability measures for functional connectivity

To evaluate temporal variability, we adopted conventional “stability” indices of FC and pFC networks using two types of measures; (1) intra-class correlation (ICC) for univariate stability of edge and (2) Pearson correlation coefficient for a similarity measure of multivariate patterns. In the correlation analyses with SC, ICC, and Pearson correlation coefficient were Fisher's r-to-z transformed to improve normality.

#### Intra-class correlation (ICC)

We measured within-a-day stability (= 1—variability) on each functional edge using within-subject variance ($\sigma_{w}^{2}$), separated from between-subject variance ($\sigma_{b}^{2}$), and ICC (Friedman L. et al., 2008; Caceres et al., 2009; Deuker et al., 2009), across eight different sessions. Two types of variances were estimated in a two-way mixed effect model and ICC was calculated using two variances in the model (Caceres et al., 2009) such as

$$\begin{array}{l} ICC=\frac{\sigma_{b}^{2}}{\sigma_{b}^{2}+\sigma_{w}^{2}}=\frac{MSB-MSE}{MSB-\left(k-1\right)MSE} \end{array}$$

where *MSB* and *MSE* represent mean squares of between- and within-subject factors, and *k* represents the number of sessions. ICC differs from repeated measures analysis of variance (ANOVA) testing *F*_0_ = *MSJ*/*MSE* where *MSJ* represents the mean squares of between-sessions factors (herein, the time-of-day). Results of the ICC were empirically interpreted as (1) highly variable, ICC < 0.4; (2) fairly variable, 0.4 ≤ ICC < 0.5; or (3) highly stable, ICC ≥ 0.5 in accordance with our previous work (Park et al., 2012).

#### Similarity for multivariate edge involvement patterns

In order to evaluate the variability of subnetwork patterns, we defined the within-a-day similarity (= inverse of variability) of the multivariate patterns. For each subnetwork (edges belonging to each edge type), all edges in the subnetwork comprised a functional connectivity vector within each session for each individual. The similarity of the multivariate edge involvement was defined as the average value of Pearson correlation coefficients between functional connectivity vectors of all pairs of eight sessions (see Figure 1B).

### Structural constraints on the variability of the functional connectivity and variability of multivariate edge involvement patterns

1. The Relationship between Structure Connectivity and Functional Connectivity Variability according to Structural Edge Types: In order to explore the relationship between structural connectivity and the variability of functional connectivity according to structural edge types, we calculated a Pearson correlation coefficient between the ICC of functional connectivity (i.e., FC and pFC) and the fiber counts across edges at edges in each structural edge type (intra-hemispheric, homologous/heterologous inter-hemispheric edges, and the rich club, feeder, and local edges in the rich club architecture). In this evaluation, we used structural connectivity averaged at a group level to define different edge types for the evaluation of functional connectivity in a common space and to compare structural connectivity with the group summary statistic (such as ICC) for functional connectivity. Therefore, fiber counts used in the current study indicate group averages of log-transformed fiber counts in the individual space. Similarly, the (average) strength of functional connectivity indicates functional connectivity averaged over the sessions and the subjects.
2. The Relationship between Structure Connectivity and FC Variability according to Pseudo and Direct Functional Edges: To explore the variability of pseudo-FC, we evaluated a Pearson correlation coefficient between the ICC of FC and fiber counts across edges over the group-level pseudo functional edges and over the direct functional edges. Pseudo functional edges in the group level were defined to be the edges that more than half of the group have FC without pFC. Meanwhile, direct functional edges in the group level were defined to be the edges that more than half of the group have both FC and pFC.
3. Variability of Multivariate Edge Involvement Patterns: We evaluated the variability (= 1—similarity) of the multivariate edge involvement patterns of FC and pFC for rich club edges (**Figure 6A**). The variability of feeder edge involvement patterns was also examined for each rich club node (**Figure 6A**). We also evaluated the variability of the multivariate edge involvement patterns of FC within a subnetwork of pseudo functional edges and within a subnetwork of direct functional edges.

## Results

### Functional variability according to structural edge types

**Figure 2:**
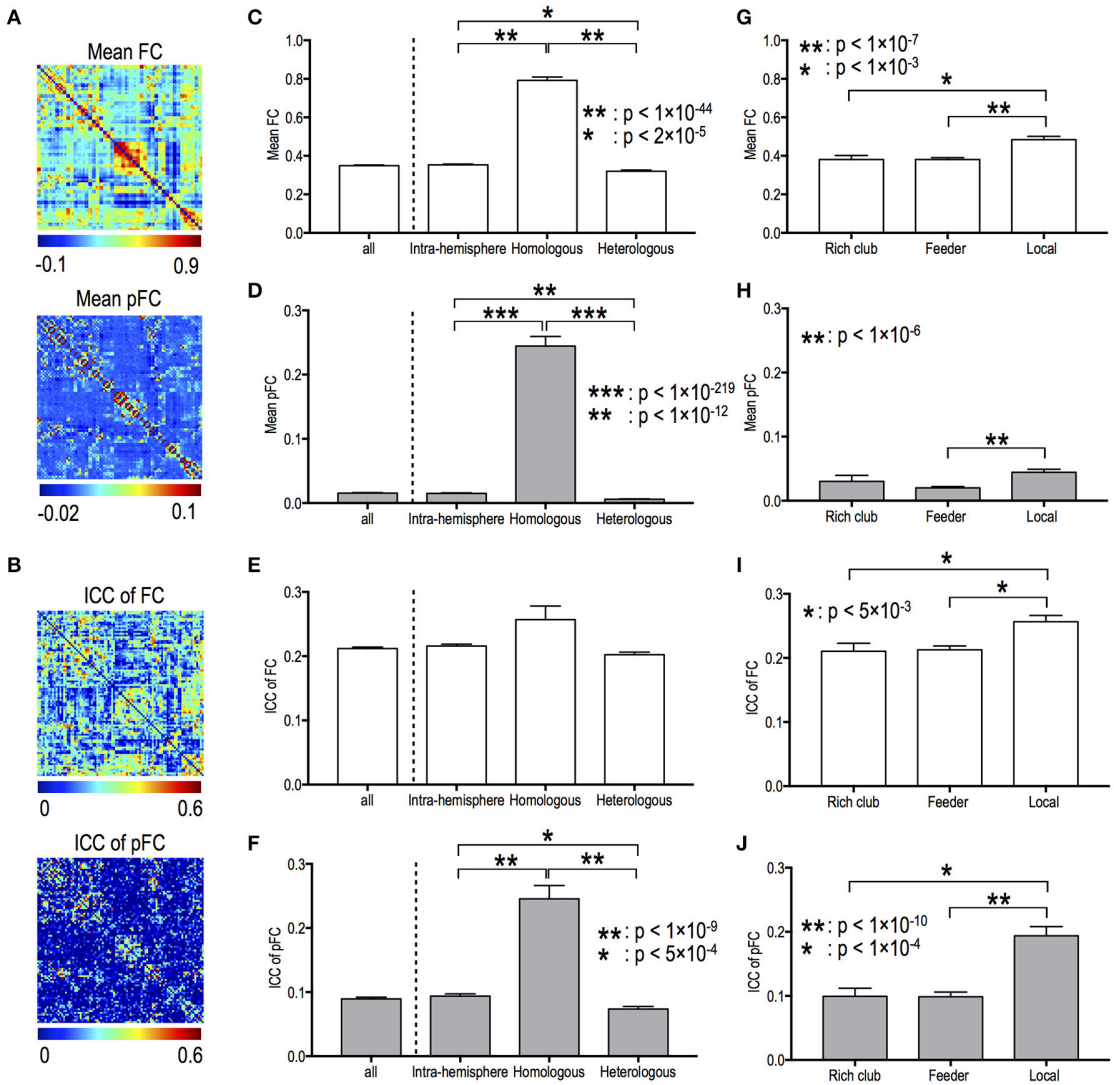
Mean functional connectivity strength and its stability according to edge types. Spatial patterns of mean functional connectivity **(A)** and of functional connectivity stability (i.e., ICC) **(B)**. Mean and ICC of FC and pFC according to the inter- and intra-hemispheric edge types **(C–F)** and according to topological edge types in rich-club organization **(G–J)**. FC: Pearson correlation-based functional connectivity, pFC: partial correlation-based functional connectivity.

Figures 2A,B present the mean functional connectivity and ICC patterns for FC and pFC networks. Mean functional connectivity and stability of functional connectivity differed according to edge types in both FC and pFC. The edge type dependency was grossly similar between FC (Figures 2C,E,G,I) and pFC (Figures 2D,H,F,J). For example, the inter-hemispheric homologous edges showed the highest mean functional connectivity in both FC and pFC (Figures 2C,D). Significant differences in mean functional connectivity were detected between intra-hemispheric and inter-hemispheric homologous edges (FC: *p* = 1.6 × 10^−45^, pFC: *p* = 6 × 10^−220^), between intra-hemispheric and inter-hemispheric heterologous edges (FC: *p* = 1.7 × 10^−5^, pFC: *p* = 4 × 10^−13^), between inter-hemispheric homologous and heterologous edges (FC: *p* = 4.7 × 10^−51^, pFC: *p* = 1 × 10^−283^), and between feeder and local edges (FC: *p* = 4 × 10^−8^, pFC: *p* = 1 × 10^−7^) in both FC (Figures 2C,G) and pFC (Figures 2D,H).

For both FC and pFC, ICC of functional connectivity showed significant difference between feeder and local edges (FC: *p* = 2 × 10^−4^, pFC: *p* = 9 × 10^−11^), and between rich club and local edges (FC: *p* = 4 × 10^−3^, pFC: *p* = 1 × 10^−5^) (Figures 2I,J). However, pFC only showed significant difference in ICC between intra-hemispheric and inter-hemispheric homologous edges (*p* = 2 × 10^−10^), between intra-hemispheric and inter-hemispheric heterologous edges (*p* = 4 × 10^−4^), and between inter-hemispheric homologous and heterologous edges (*p* = 1 × 10^−18^) (Figure 2F).

**Figure 3:**
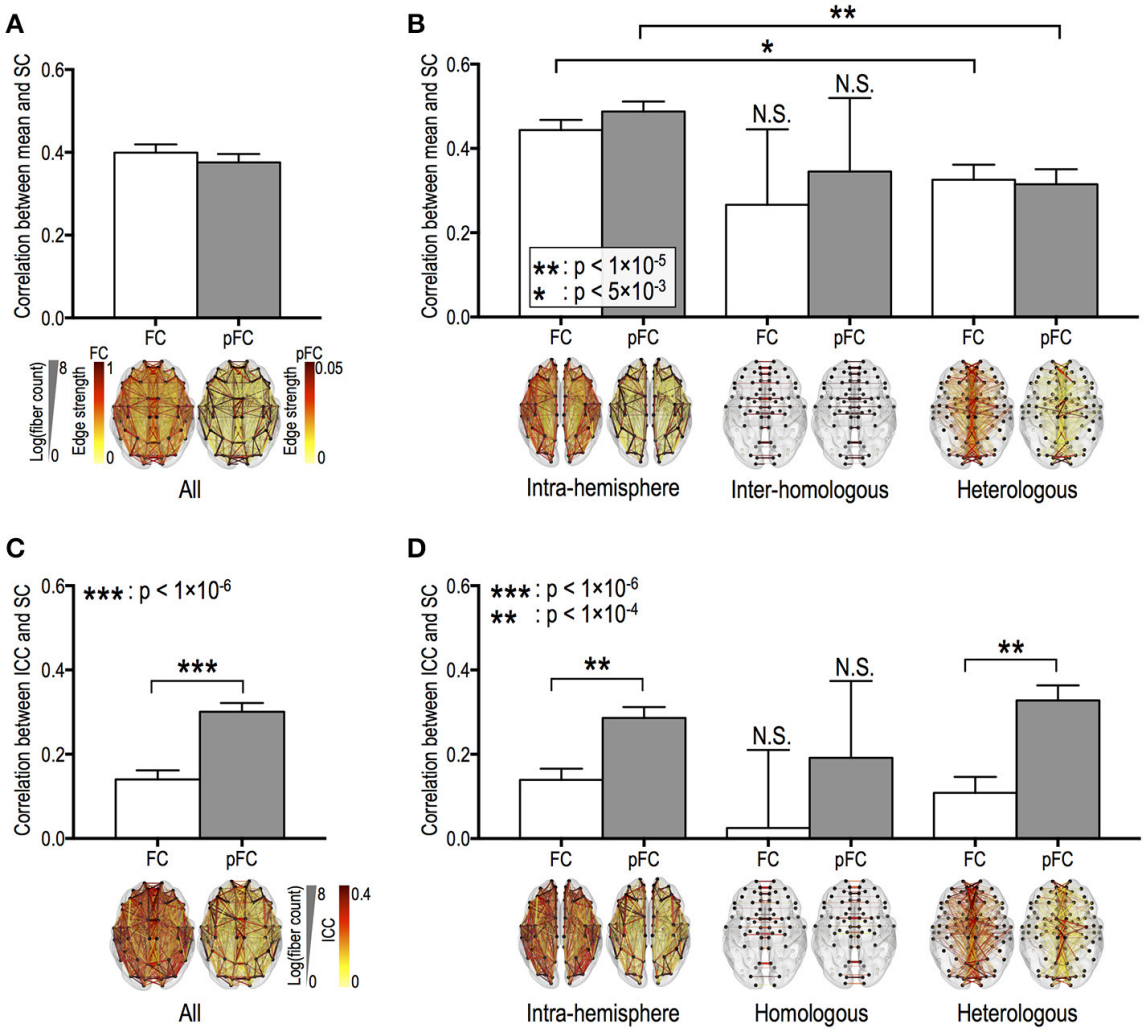
Relationships between the stability of functional connectivity and structural connectivity according to edge types. The relationships between functional connectivity strength and structural connectivity (SC, defined by log-transformed fiber counts) for whole brain edges **(A)** and for intra-hemispheric edges and for inter-hemispheric homologous/heterologous edges **(B)**. The relationships between intra-class correlations (ICCs, stability) of functional connectivity and log-transformed fiber counts in the different edge types **(C,D)**. FC: Pearson correlation-based functional connectivity, pFC: partial correlation-based functional connectivity. N.S. represents “no significant difference.”

Fiber counts were positively correlated with strengths of FC and pFC in all edge types (Figures 3A,B), except for homologous inter-hemispheric edges (Figure 3B), where significantly higher in the intra-hemispheric edges than in the heterologous inter-hemispheric edges (*p* < 5 × 10^−3^) (Figure 3B). These positive relationships were similarly found in FC and pFC. However, fiber counts show positive correlation with stability (i.e., ICC) in pFC more than in FC (Figure 3C). More specifically, functional connectivity in an edge with a higher number of fiber bundles showed significantly higher ICC in both pFC (*r* = 0.3, *p* = 4 × 10^−45^) and FC (*r* = 0.14, *p* = 1 × 10^−10^) and the relationship was much stronger in pFC than in FC (*p* = 1 × 10^−15^) (Figure 3C). Such stronger positive relationship in ICC of pFC was consistently found in intra-hemispheric edges (*r* = 0.29, *p* = 3 × 10^−27^) and heterologous inter-hemispheric edges (*r* = 0.33, *p* = 6 × 10^−19^) but not in homologous inter-hemispheric edges (Figure 3D).

ICC of pFC was positively correlated with fiber length only in heterologous inter-hemispheric edges (*r* = 0.15, *p* = 1 × 10^−4^). Meanwhile, ICC of FC show no relationship with fiber lengths in intra-hemispheric edges and inter-hemispheric edges (*p* > 0.005; Bonferroni correction across 10 tests was applied with *p* < 0.05). Homologous inter-hemispheric edges did not show any relationship between ICC and both fiber counts and lengths in both FC and pFC.

### Variability of functional connectivity in pseudo and direct functional edges

**Figure 4:**
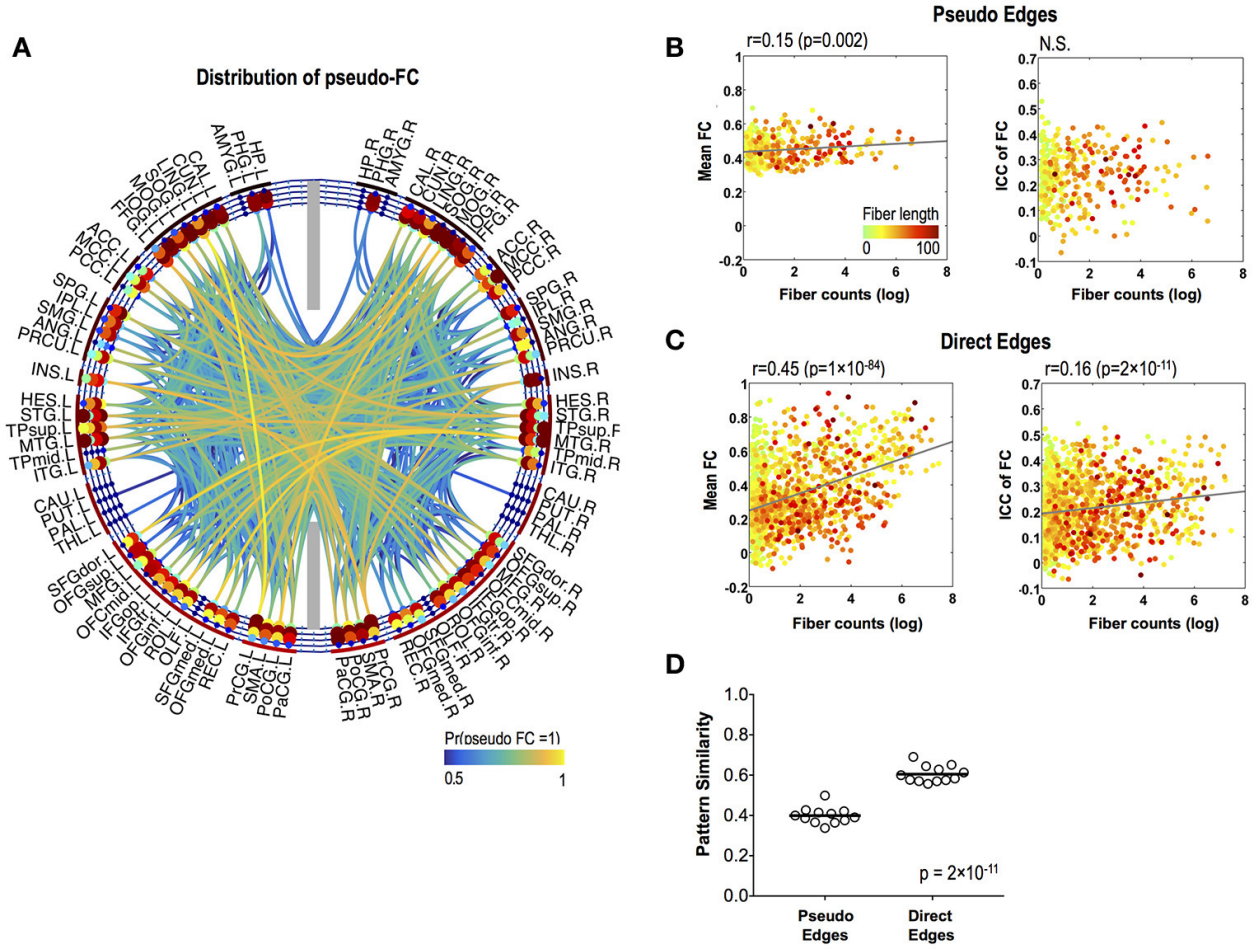
Stability of pseudo-FC. **(A)** The spatial distribution of pseudo-FC. The relationships between the mean functional connectivity of FC and log-transformed fiber counts (left) and between the stability (ICC) of FC and log-transformed fiber counts (right) for pseudo edges **(B)** and direct edges **(C). (D)** Pattern similarity of FC within a subnetwork of pseudo functional edges and within a subnetwork of direct functional edges. The dots in **(B,C)** are colored according to the fiber lengths where we set greater than 100 to 100 mm for effective color-coding since such connections were very rare in our data.

Pseudo functional edges were mostly found in the temporal lobe (Figure 4A). Fiber counts showed significantly lower positive correlation with mean strength of FC in pseudo functional edges than in direct functional edges (*p* = 4 × 10^−16^) (Figures 4B,C). Fiber counts were also positively correlated with ICC of FC in direct functional edges (*r* = 0.16, *p* = 2 × 10^−11^) and the relationship did not appear in pseudo functional edges (Figures 4B,C). FC patterns showed higher variability (=lower pattern similarity) across sessions in pseudo functional edges than in direct functional edges (*p* = 2 × 10^−11^) (Figure 4D).

### Variability according to topological edge types in rich club structures

**Figure 5:**
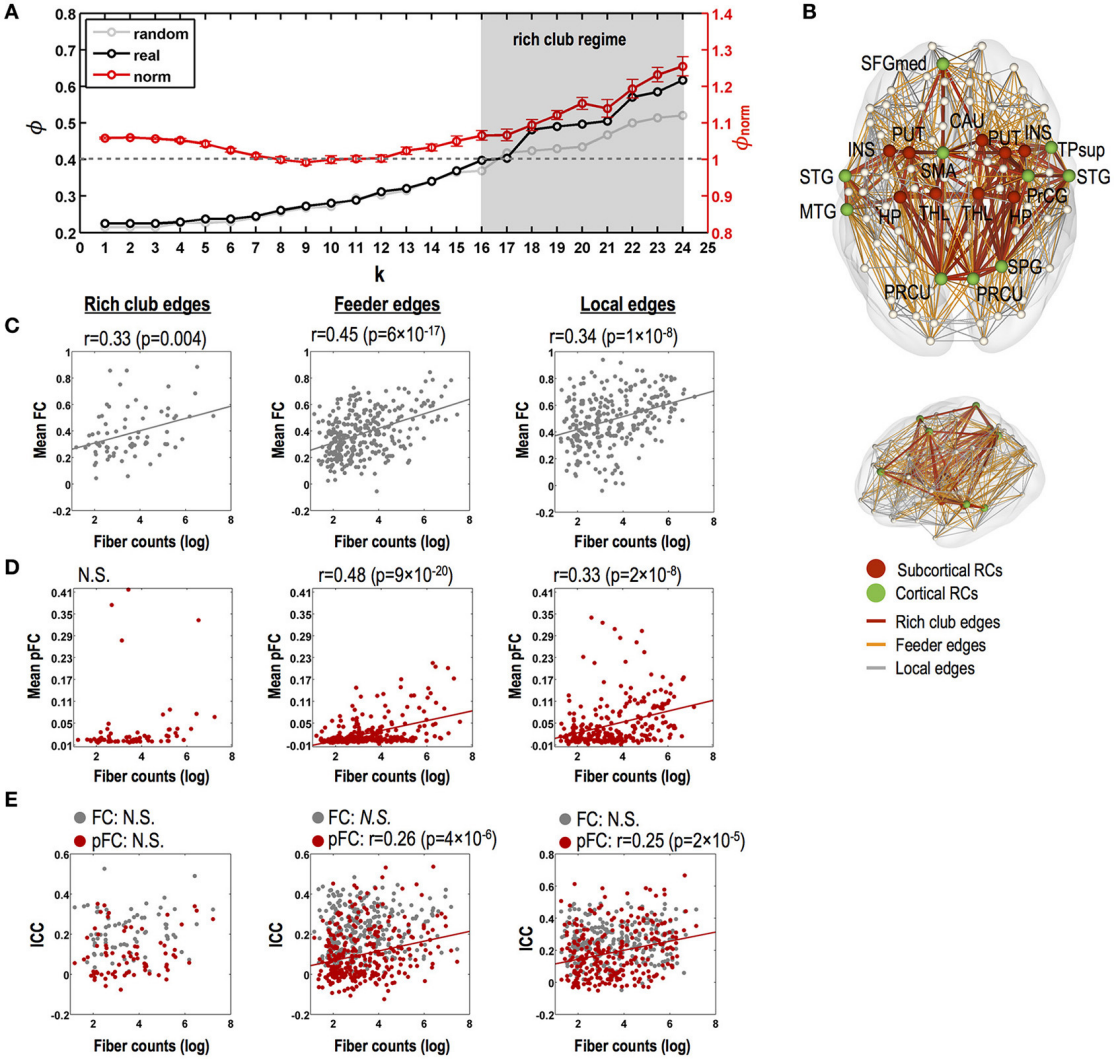
Relationships between stability of functional connectivity and structural connectivity according to topological edge types in rich club organization. **(A)** Significant rich club organization in the range of degree thresholds, 16 ≤ k ≤ 24 (gray shade; *P* < 0.05, Bonferroni corrected). Black line indicates time series of group-averaging rich club coefficients with changing degree threshold of k. Light gray line indicates time series of mean rich club coefficients for 1,000 random networks and red line and its error bar represent mean normalized rich club coefficients and its standard error of mean. **(B)** Rich club nodes by a degree threshold of *k* = 18 and three kinds of structural edges (i.e., rich club, feeder, and local edges). **(C)** Relationships between strength of FC and log-transformed fiber counts in rich club, feeder, and local edges for FC. **(D)** Relationships between strength of pFC and log-transformed fiber counts in rich club, feeder, and local edges for FC. **(E)** Relationships between stability (ICC) and strength of FC (gray circles lines) and pFC (red circles and regression lines) and log-transformed fiber counts in rich club, feeder, and local edges. N.S. indicates no significance. THL, thalamus; PUT, putamen; PRCU, precuneus; HP, hippocampus; SFGmed, medial superior frontal gyrus; PrCG, precentral gyrus; STG, superior temporal gyrus; INS, insula; SPG, superior pariental gyrus; TPsup, superior temporal pole; CAU, caudate; L, left; R, right.

Our structural network data show a rich club-like organization with degree thresholds of k = 16 to k = 24, where normalized coefficients are significantly greater than 1 (*p* < 0.05, Bonferroni corrected) (Figure 5A). In constructing rich clubs, we choose a degree threshold of k = 18 since it showed the most significant value (*p* = 7 × 10^−5^). We found 19 rich club nodes (degree threshold of k = 18, Figure 5B); the bilateral putamen, thalamus, insula, hippocampus, and precuneus and the left superior frontal and right superior parietal gyrus, all of which are areas supported by previous studies (van den Heuvel and Sporns, 2011; Kim et al., 2014), and the bilateral superior temporal gyrus, left middle temporal gyrus, left supplementary motor area, right superior temporal pole, right precentral gyrus, and right caudate.

No relationship was found between fiber counts and ICC of FC in all types of rich club edges, feeder edges, and local edges (Figure 5C). Although ICC of pFC was not correlated with fiber counts in rich club edges, ICCs of pFC were significantly correlated with fiber counts in the feeder edges (*r* = 0.26, *p* = 4 × 10^−6^) and local edges (*r* = 0.25, *p* = 2 × 10^−5^) without a statistical difference between these two correlations. Also, the strength of FC and pFC showed significant positive correlations with fiber counts in feeder edges (FC: *r* = 0.45, *p* = 6 × 10^−17^; pFC: *r* = 0.48, *p* = 9 × 10^−20^; no difference between two) and local edges (FC: *r* = 0.34, *p* = 1 × 10^−8^; pFC: *r* = 0.33, *p* = 2 × 10^−8^; no difference between two) (Figures 5D–E).

**Figure 6:**
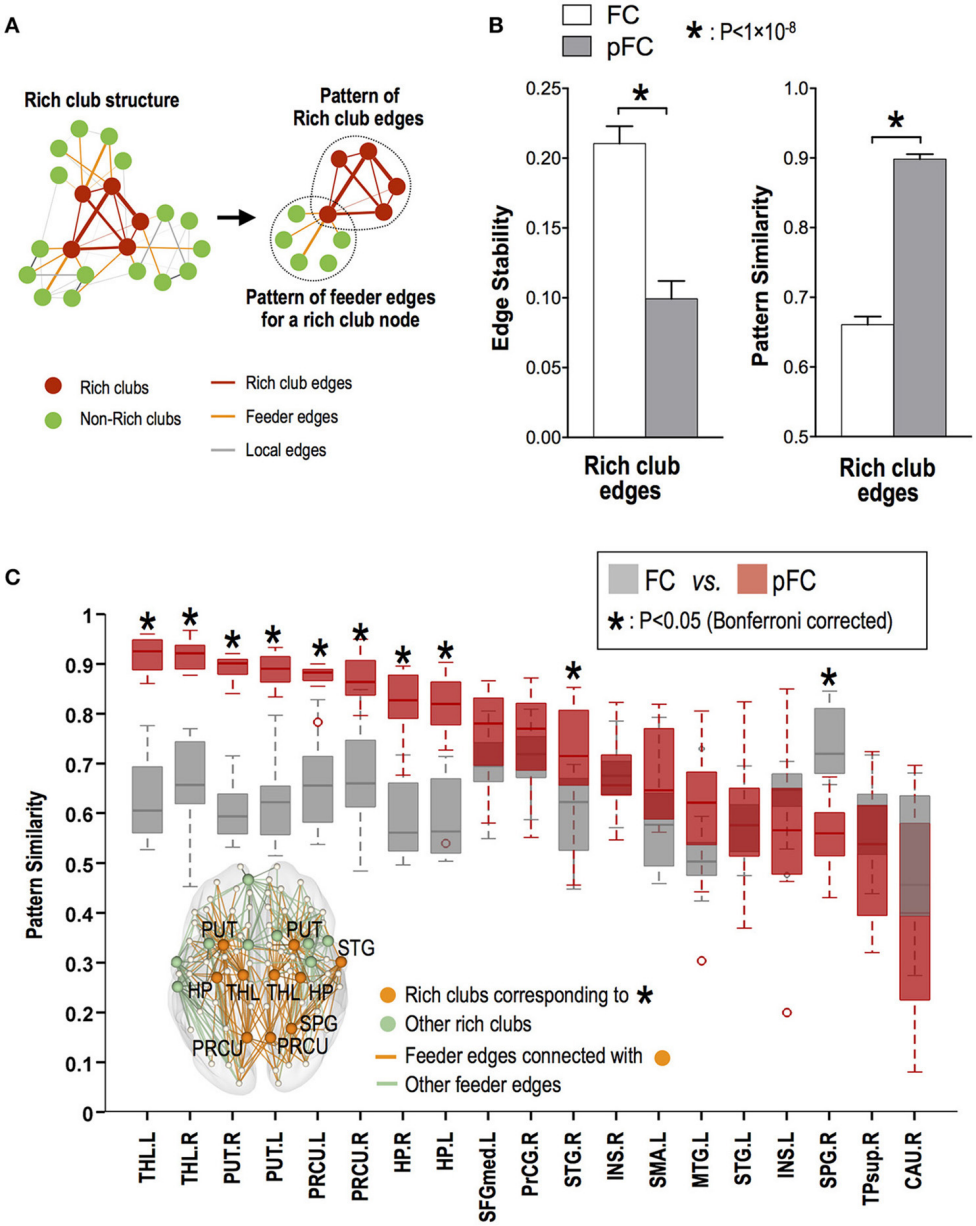
Stability of multivariate edge involvement patterns for rich club and feeder edges. **(A)** Multivariate edge involvement pattern in a subnetwork composed of rich club edges and the pattern in a subnetwork composed of feeder edges for each rich club node. **(B)** Univariate edge stability (mean ICC) of functional connectivity and temporal similarity of multivariate edge involvement patterns for rich club edges. **(C)** Stability of feeder edge involvement patterns for a subnetwork of each rich club node, where star (^*^) indicates significant difference between pattern stability in FC and stability in pFC. THL, thalamus; PUT, putamen; PRCU, precuneus; HP, hippocampus; STG, superior temporal gyrus; SPG, superior parietal gyrus; L, left; R, right.

While rich club edges showed significantly higher stability (ICC) of functional connectivity in FC than in pFC (*p* < 0.05, Bonferroni corrected), multivariate similarity analysis of edge patterns (or subnetworks) in rich club edges showed higher stability in pFC than in FC (*p* = 2 × 10^−10^) (Figure 6B).

Among rich club nodes, the bilateral thalamus, putamen, precuneus, and hippocampus and right superior temporal gyrus showed more stable multivariate patterns of feeder functional connectivity in pFC than in FC (*p* < 0.05, Bonferroni corrected) (Figure 6C).

## Discussion

By evaluating the variability of functional connectivity over the course of “several hours within a single day”, we found that structural connectivity generally constrained both the strength and the variability of functional connectivity and multivariate edge involvement patterns, particularly for sparse direct functional connectivity (i.e., pFC), within a day. More specifically, the structural constraints on the variability of functional connectivity differed according to structural edge types, differentiating heterologous inter-hemispheric and intra-hemispheric edges from homologous inter-hemispheric edges, and feeder and local edges from rich club edges.

### Variability of p*FC* depends strongly on fiber counts except for homologous inter-hemispheric edges and rich club edges

In accordance with previous studies (Honey et al., 2009; Hermundstad et al., 2013), we found that the structural connectivity (particularly fiber counts) constrained the “strengths” of both FC and pFC. We further revealed strong structural constraints on the within-a-day “variability” of functional connectivity, which were more prominent in pFC than FC and differed across different edge types.

Except for stable homologous inter-hemispheric edges, fiber counts generally affected the variability of pFC, as well as the strength of pFC, across time within a single day. Similarly, the variability of pFC at feeder edges and local edges under the rich club topology was constrained by the fiber counts. In those edges, a larger number of fiber bundles sustains a more stable level of direct functional connectivity across time points in a single day. Conversely, edges that were supported by a smaller number of fibers showed higher variability. This strong dependency of within-a-day variability of pFC on fiber counts is consistent with previous studies on the relationship between “micro-state variability” of FC and fiber counts (Zalesky et al., 2014; Liao et al., 2015; Zhang et al., 2016), as explained in Introduction.

Fiber lengths generally showed no relationship with the variability of pFC except for a very weak relationship in the heterologous inter-hemispheric edges. These findings are similar to previous studies (Misic et al., 2014; Shen et al., 2015a,b), where the variability of functional connectivity was associated with fiber counts but not with inter-regional distance.

### Variability of pFC is independent of fiber counts in homologous inter-hemispheric edges and rich club edges

In contrast to the intra-hemispheric edges and heterologous inter-hemispheric edges, homologous inter-hemispheric edges did not show any relationship between fiber counts and the variability of functional connectivity in both pFC and FC. Homologous inter-hemispheric edges, which correspond to dense callosal fibers (Hofer and Frahm, 2006; Jarbo et al., 2012), have a stronger structural basis compared with other types of edges (Shen et al., 2015b). However, fiber counts did not proportionally reduce the pFC variability in these dense structural edges. Similarly, rich club edges, which have strong structural connectivity among rich club nodes, did not show a significant correlation between pFC variability and fiber counts. Both homologous inter-hemispheric edges and rich club edges have highly dense fibers (high structural connectivity). Within these dense edges, the variability of functional connectivity was not proportionally constrained by fiber counts. It is possible that fibers in a dense edge may not be homogeneous and may lead to dynamic variations of functional connectivity among pairs of multiple subregions in the two regions that the dense edge interconnects (Park et al., 2013; Gordon et al., 2016). Thus, in edges with strong structural connectivity, the variability of functional connectivity may not only be regulated by fiber counts but also depend on some other factors such as a functional nodal composition (subcomponents) and functional role of each node in the brain. Therefore, the relationship between structural connectivity and variability of functional connectivity is more than a simple generalization that higher structural connectivity leads to lower temporal variation of functional connectivity. However, the details remain to be explored in the future study.

### FC diverges from pFC in the dependency of variability on structural connectivity

The “strengths” of both FC and pFC were highly constrained by the SC (fiber counts). However, the structural constraint on the “variability” of functional connectivity was only significant in pFC, not in FC (Figure 3C). This is prominent in intra-hemispheric and heterologous inter-hemispheric edges. Although no significant correlation was found between the variability of FC and SC (fiber counts) in all of the edge types (rich club, feeder, and local edges), the variability of pFC (but not FC) at feeder edges and local edges was constrained by the fiber counts.

The divergence of FC from pFC was manifested in the pseudo functional edges (non-zero FC edges over zero pFC), the characteristics of which were firstly explored in this study. Although the mean functional connectivity strength in the pseudo functional edges showed a weak relationship with fiber counts, the within-a-day variability did not show any relationship with fiber counts, which contrasted with the functional connectivity in direct functional edges (where showed significant correlations with fiber counts). Furthermore, pseudo functional edges exhibited highly variable FC patterns across sessions within a day, compared to direct functional edges. These results suggested that pseudo functional edges dynamically emerge according to various neural contexts possibly through polysynaptic pathways or common modulation.

### Variability of the multivariate edge involvement patterns for subnetworks

A particular brain function consists of a dynamic congregation of edges and a single edge may be dynamic as it involves dynamic brain functions at different time points. However, the current result implies that the patterns of how single edges congregate together to compose dynamic functions are stable. This is supported by the extraction of common edge patterns from the resting state fluctuations (Park et al., 2014).

Most individual edges in the rich club organization showed dynamic FC and pFC across times within a day. While each edge had higher variability in pFC than in FC, the variability of multivariate edge involvement patterns of pFC was significantly lower than that of FC. In particular, the edge involvement patterns of pFC in the rich club edges across the time samples were very stable, despite the high univariate variability of pFC at each rich club edge (Figure 6B). Meanwhile, FC showed reverse directions, relatively low univariate variability but high multivariate variability. Considering the “degeneracy” of the brain system (Edelman and Gally, 2001; Price and Friston, 2002), the relatively stable FC in each edge might be composed of different configurations of dynamic direct interactions (pFC). This divergence of FC from pFC was clearly seen in the patterns of the feeder edges for rich club nodes in the thalamus, putamen, precuneus, and hippocampus. Those rich club nodes showed more stable feeder connectivity patterns of pFC than cortical rich club nodes (Figure 6C). These results imply that the pFC variations depend on the topological role of the edge and are modulated by the different levels of structural connectivity.

The variability of the functional network is an increasingly important issue, as functional brain networks are widely used to characterize individual personality (Barnes et al., 2014; Finn et al., 2015) and identify brain diseases (Smith et al., 2009; Fox and Greicius, 2010; Laird et al., 2013; Sadaghiani and Kleinschmidt, 2013; Stam, 2014). Thus far, the variability of functional networks has been shown mainly using a multivariate approach, for example, the correlation of elements in the functional connectivity matrix (Finn et al., 2015), global network properties (Bullmore and Sporns, 2009, 2012) of the “Pearson-based correlation matrix” averaging-out within a session, and ICA analysis (Calhoun et al., 2001; Beckmann et al., 2005). Meanwhile, dozens of reproducibility studies have conducted test-retests of Pearson correlation-based functional network measures (e.g., Braun et al., 2012). The highly stable edge involvement patterns in the feeder edges of the pFC network (and not in the FC network) is consistent with previous studies that showed stable characteristics of multivariate functional composition (Calhoun et al., 2001; Beckmann et al., 2005).

The current results suggest that pFC is superior and more sensitively reflects the dynamic functional nature of the whole brain network compared with FC.

### Methodological issues

In brain network studies, the inclusion of a large number of brain regions, relative to a conventional number of observations, may prevent reliable estimation of partial correlation, which can be solved using regularization techniques reducing very small edge values to zero (Friedman J. et al., 2008; Huang et al., 2010; Varoquaux et al., 2010; Smith et al., 2011). Thus, the regularization in a partial correlation approach is suitable in most fMRI-based brain network studies, especially when applying a sliding window approach to investigate brain network dynamics with a small number of observations (Cribben et al., 2012; Allen et al., 2014; Calhoun et al., 2014; Monti et al., 2014). In order to apply such regularization, it is required to select the regularization amount as a parameter, which is related to network sparsity. In this study, we used a criterion, StARS, which selects an optimal regularization amount, giving more reliable and sparse network based on random sampling (Liu et al., 2010). StARS estimates more accurately and more similar sparsity to the true network than AIC, BIC, and cross-validation approaches (Liu et al., 2010). However, it should be noted that the partial correlation approach, despite the sparsity estimation strategy, may underestimate the real connectivity in the brain, which should not be ignored in the interpretation of the results using this method.

In spite of potential inter-individual variations in structural connectivity, we used the group-averaged structural connectivity (averaged across 12 subjects) to associate it with the average strength of the functional connectivity (averaged across total of 12 subjects and 8 sessions) or variability of the functional connectivity defined using ICC in the group level. This makes it possible to evaluate dynamic functional connectivity in a common space (particularly for the sparse connectivity) and to compare structural connectivity with the group summary statistic (such as ICC) for functional connectivity. It mitigates the missing fibers or false alarm fibers during fiber tractography in the individual space.

Compared to FC, pFC was generally more variable than FC for all edge types. It is possible that pFC may reflect dynamic nature of the brain connectivity more than FC. However, we cannot disregard the possibility arisen from the characteristic of pFC estimation, which utilizes non-linear shrinkage around zero. This non-linear shrinkage makes it difficult to evaluate mean and variability of pFC in a way comparable to those of FC (a continuous metric). Accordingly, we evaluated variability of the edge by associating with structural connectivity rather than comparing mean strength and variability of pFC with FC.

The current study was conducted with fMRI signals without global signal regression (GSR). Since the effects of GSR on functional connectivity still remain controversial (Murphy et al., 2009; Chai et al., 2012), we conducted the same analysis with signals obtained after GSR and presented the results in the Supplementary Materials. Overall results and tendencies were highly similar between signals obtained with GSR and the ones obtained without GSR (Supplementary Materials). When we conducted the current evaluation with normalized structural connectivity by dividing fiber counts by mean regional volume sizes (average numbers of voxels at two regions), the results were highly consistent with the results from the current evaluation (Supplementary Materials).

The fundamental cause of variability in functional connectivity within a day is not yet fully understood. There have been several researches on the effects of sleepiness on functional connectivity (Verweij et al., 2014; Kaufmann et al., 2016; Zhu et al., 2016). Effects of the circadian rhythm or time of day may also affect variability of functional connectivity. Instead of considering those factors as unwanted signals, we regard those factors as potential sources of within-a-day variability of functional connectivity. However, we could not control for the confounding factors inevitable in the data acquisition in the laboratory setting, which is different from everyday environment.

In summary, these results show that structural connectivity generally constrains not only the strength of the functional connectivity but also the variability of the functional connectivity. The structural constraints on the variability of three different types of functional connectivity differ according to edges properties and topological edge types. The edges in the pFC network more sensitively reflect dynamic functionality, which is constrained by structural connectivity but are more stable in the pattern of congregation, compared with FC. Studies of edge involvement patterns using multivariate properties in brain networks have been important in providing a detailed understanding of how the brain works. How functional connectivity differs at specific time points within a day requires further study, and the results will expand our understanding of the time of day effects on functional brain connectivity.

## Ethics statement

All subjects gave written informed consent in accordance with the Declaration of Helsinki. The protocol was approved by the Institutional Review Board of Yonsei University Severance Hospital.

## Author contributions

Conceived and designed the experiments: HP. Performed the experiments: BP. Analyzed the data: BP. Contributed reagents/materials/analysis tools: BP and JE. Wrote the paper: BP and HP.

### Conflict of interest statement

The authors declare that the research was conducted in the absence of any commercial or financial relationships that could be construed as a potential conflict of interest. The reviewer XL and handling Editor declared their shared affiliation, and the handling Editor states that the process nevertheless met the standards of a fair and objective review.
