# Supplementary material for: Structural Brain Connectivity Constrains within-a-Day Variability of Direct Functional Connectivity
Source: Front Hum Neurosci. 2017 Aug 10;11:408. doi: 10.3389/fnhum.2017.00408 (PMC5554338; doi:10.3389/fnhum.2017.00408)
Supplement: Supplementary file 1 [file DataSheet1.PDF]

Supplementary Materials

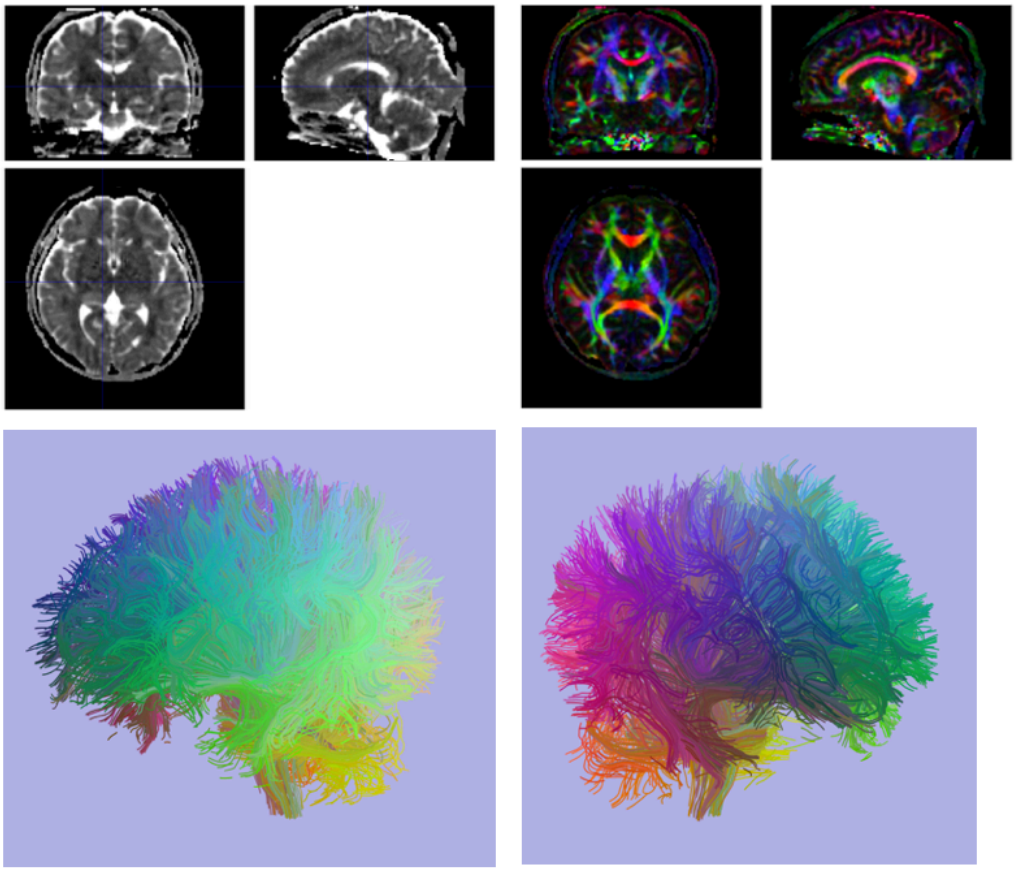

Figure S1. A DTI example of a subject. Coronal, sagittal and axial slices of a DWI volume (left top panel), a map of primary eigenvectors of DTI (colored in red, green and blue according to vector elements, right top panel), a whole brain fiber tractography (bottom panels). All images were constructed using DoDTI (Yonsei University, http://neuroimage.yonsei.ac.kr/dodti).

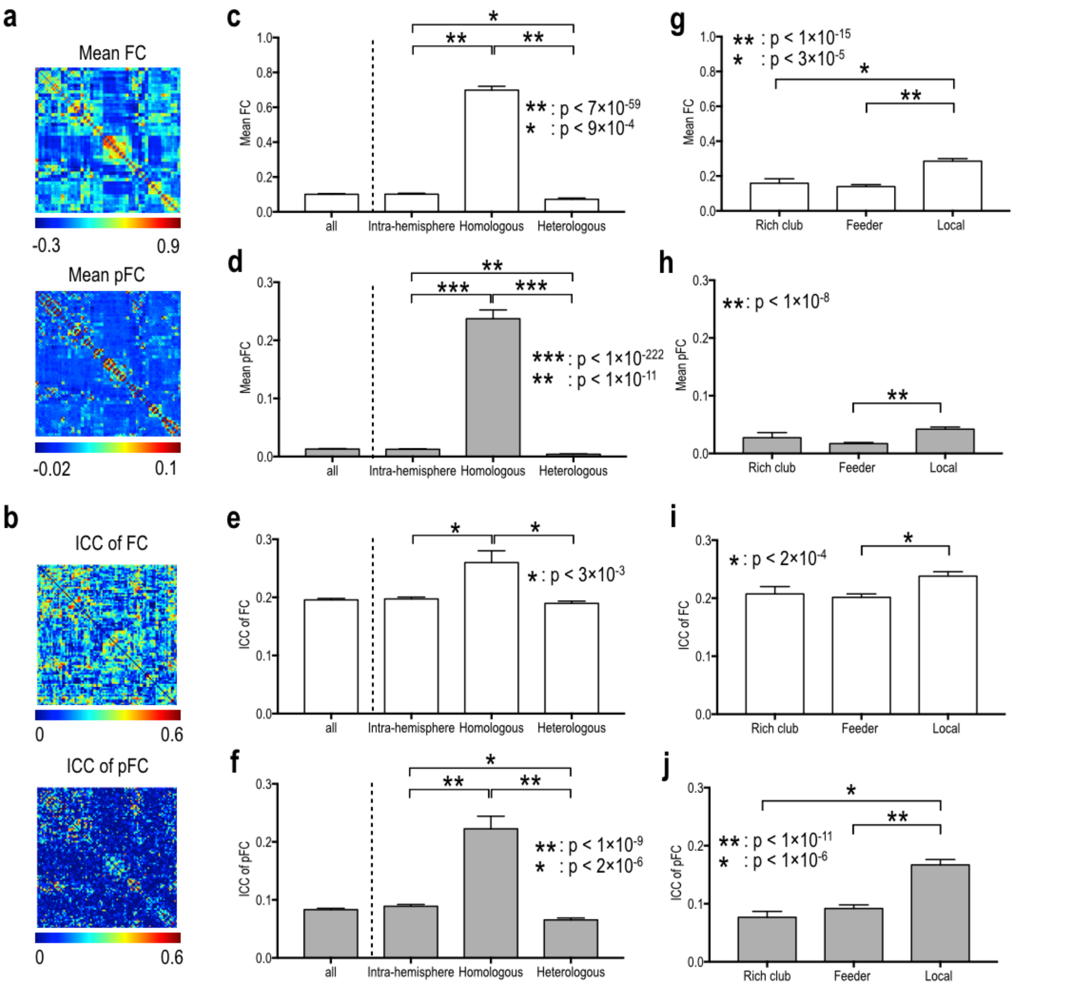

Figure S2. Mean functional connectivity strength and its stability according to edge types after global signal regression. Spatial patterns of mean functional connectivity (a) and of functional connectivity stability (i.e., ICC) (b). Mean and ICC of FC and pFC according to the inter- and intra-hemispheric edge types (c-f) and according to topological edge types in rich-club organization (g-j). FC: Pearson correlation-based functional connectivity, pFC: partial correlation-based functional connectivity.

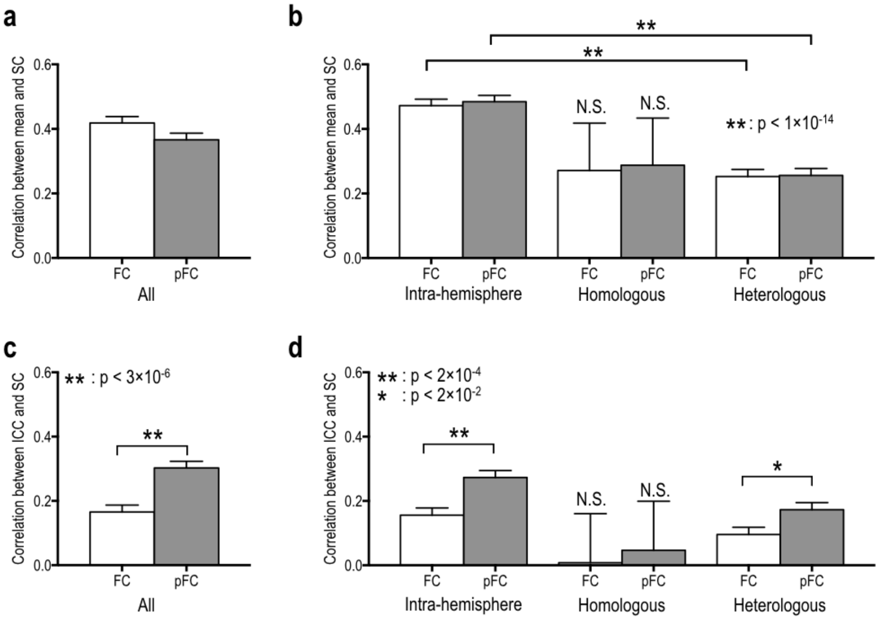

Figure S3. Relationships between the stability of functional connectivity and structural connectivity according to edge types after global signal regression. The relationships between functional connectivity strength and structural connectivity (SC, defined by log-transformed fiber counts) for whole brain edges (a) and for intra-hemispheric edges and for inter-hemispheric homologous/heterologous edges (b). The relationships between intra-class correlations (ICCs, stability) of functional connectivity and log-transformed fiber counts in the different edge types (c)-(d). FC: Pearson correlation-based functional connectivity, pFC: partial correlation-based functional connectivity. N.S. represents "no significant difference."

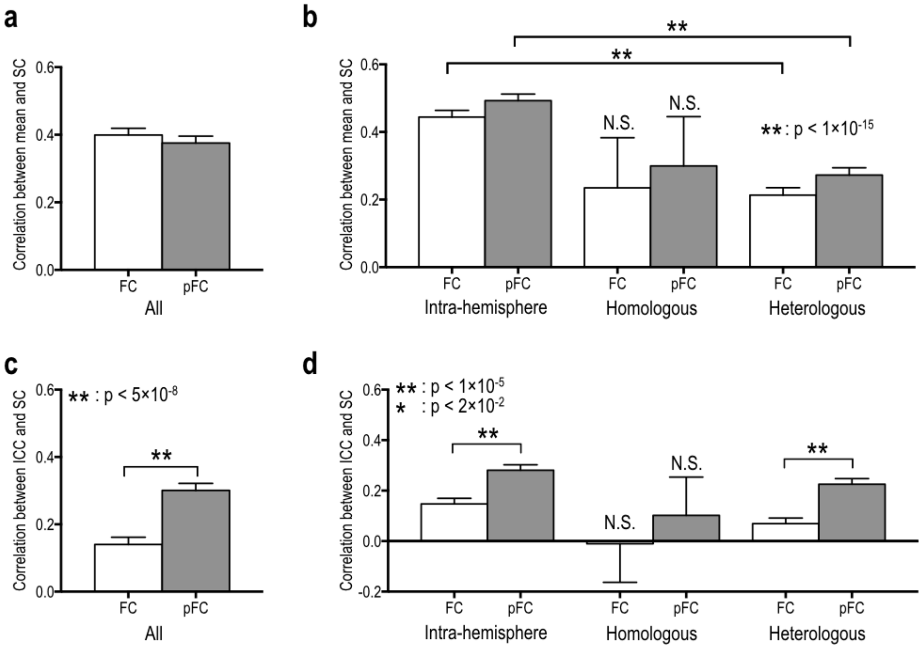

Figure S4. Relationships between the stability of functional connectivity and structural connectivity according to edge types using normalized fiber counts. The relationships between functional connectivity strength and structural connectivity (SC, defined by log-transformed fiber counts) for whole brain edges (a) and for intra-hemispheric edges and for inter-hemispheric homologous/heterologous edges (b). The relationships between intra-class correlations (ICCs, stability) of functional connectivity and log-transformed fiber counts in the different edge types (c)-(d). FC: Pearson correlation-based functional connectivity, pFC: partial correlation-based functional connectivity. N.S. represents “no significant difference.”

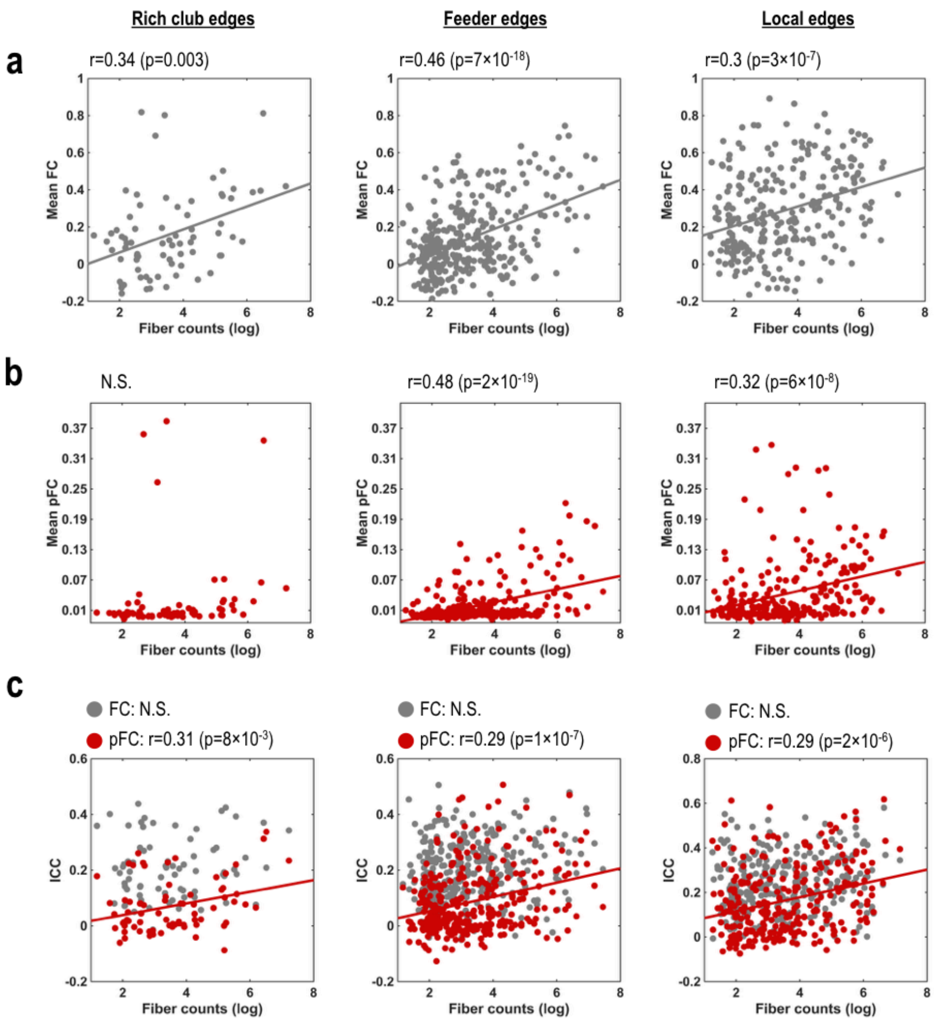

Figure S5. Relationships between stability of functional connectivity and structural connectivity according to topological edge types in rich club organization after global signal regression. (a) Relationships between strength of FC and log-transformed fiber counts in rich club, feeder, and local edges for FC. (b) Relationships between strength of pFC and log-transformed fiber counts in rich club, feeder, and local edges for FC. (c) Relationships between stability (ICC) and strength of FC (gray circles lines) and pFC (red circles and regression lines) and log-transformed fiber counts in rich club, feeder, and local edges. N.S. indicates no significance.

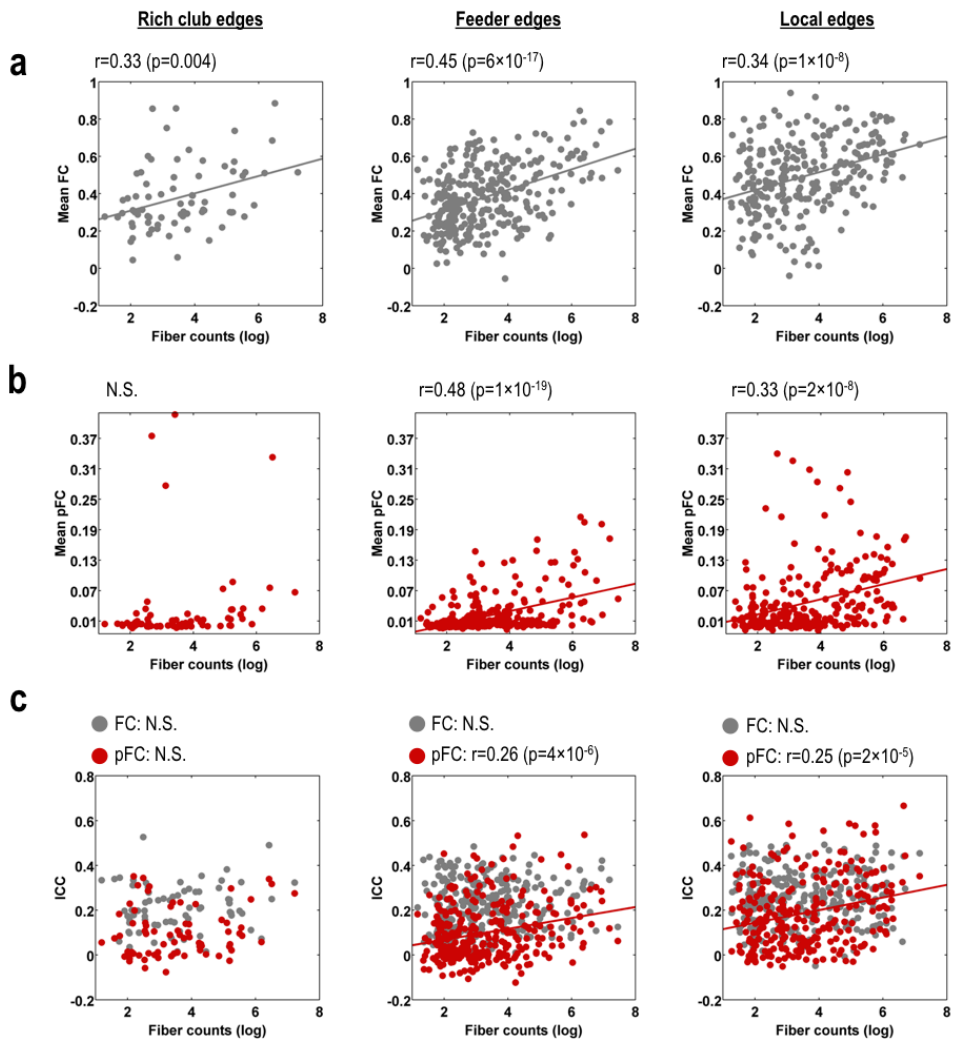

Figure S6. Relationships between stability of functional connectivity and structural connectivity according to topological edge types in rich club organization using normalized fiber counts. (a) Relationships between strength of FC and log-transformed fiber counts in rich club, feeder, and local edges for FC. (b) Relationships between strength of pFC and log-transformed fiber counts in rich club, feeder, and local edges for FC. (c) Relationships between stability (ICC) and strength of FC (gray circles lines) and pFC (red circles and regression lines) and log-transformed fiber counts in rich club, feeder, and local edges. N.S. indicates no significance.

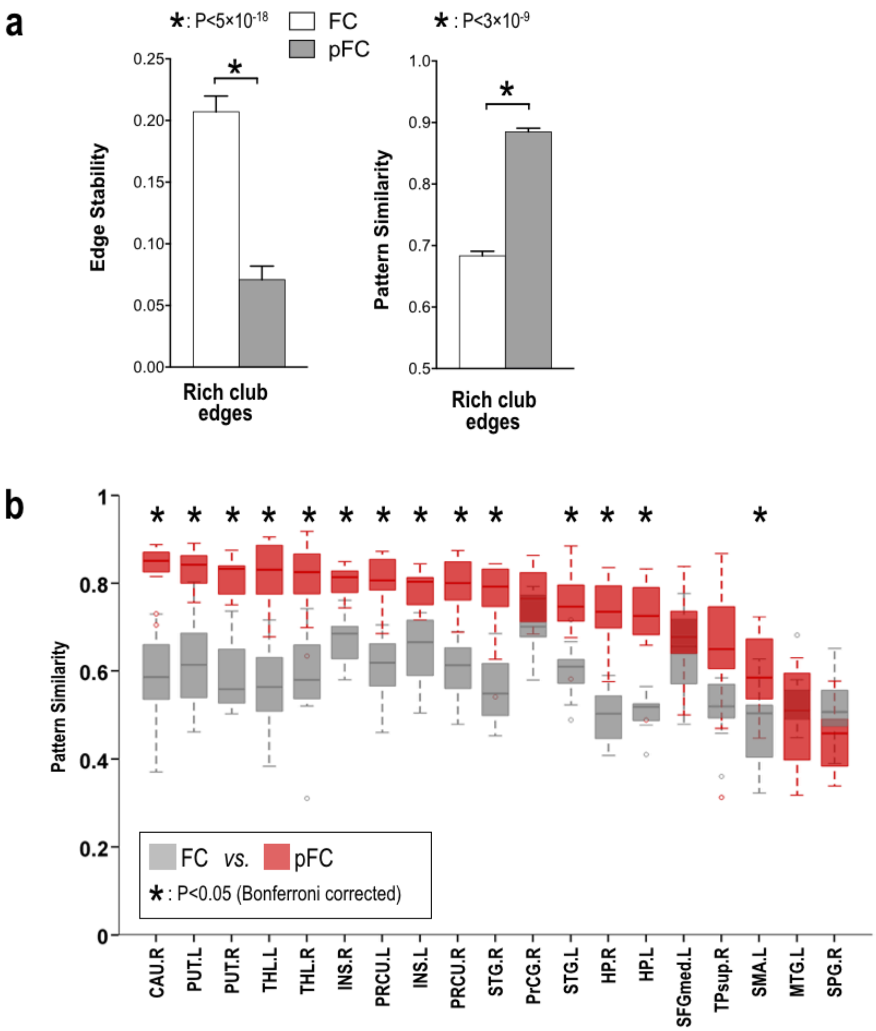

Figure S7. Stability of multivariate edge involvement patterns for rich club and feeder edges after global signal regression. (a) Univariate edge stability (mean ICC) of functional connectivity and temporal similarity of multivariate edge involvement patterns for rich club edges. (c) Stability of feeder edge involvement patterns for a subnetwork of each rich club node, where star (*) indicates significant difference between pattern stability in FC and stability in pFC. Abbreviations: THL, thalamus; PUT, putamen; PRCU, precuneus; HP, hippocampus; STG, superior temporal gyrus; SPG, superior parietal gyrus; L, left; R, right.

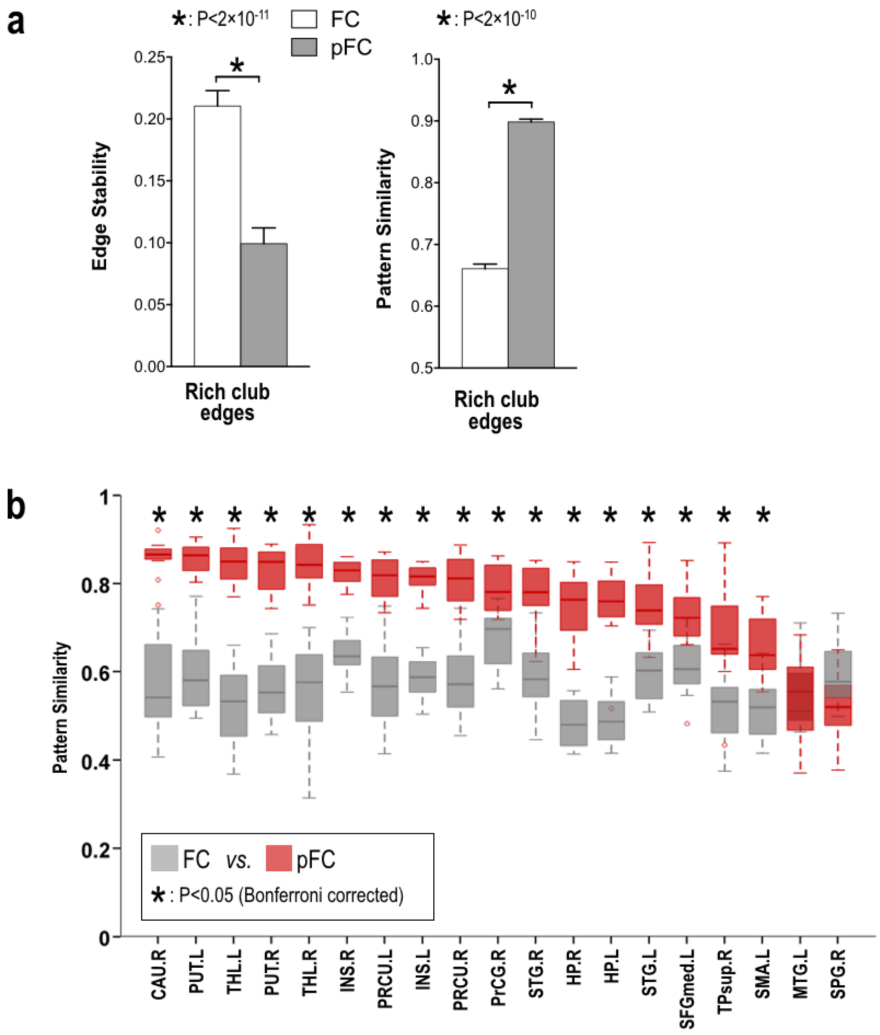

Figure S8. Stability of multivariate edge involvement patterns for rich club and feeder edges using normalized fiber counts. (a) Univariate edge stability (mean ICC) of functional connectivity and temporal similarity of multivariate edge involvement patterns for rich club edges. (c) Stability of feeder edge involvement patterns for a subnetwork of each rich club node, where star (*) indicates significant difference between pattern stability in FC and stability in pFC. Abbreviations: THL, thalamus; PUT, putamen; PRCU, precuneus; HP, hippocampus; STG, superior temporal gyrus; SPG, superior parietal gyrus; L, left; R, right.

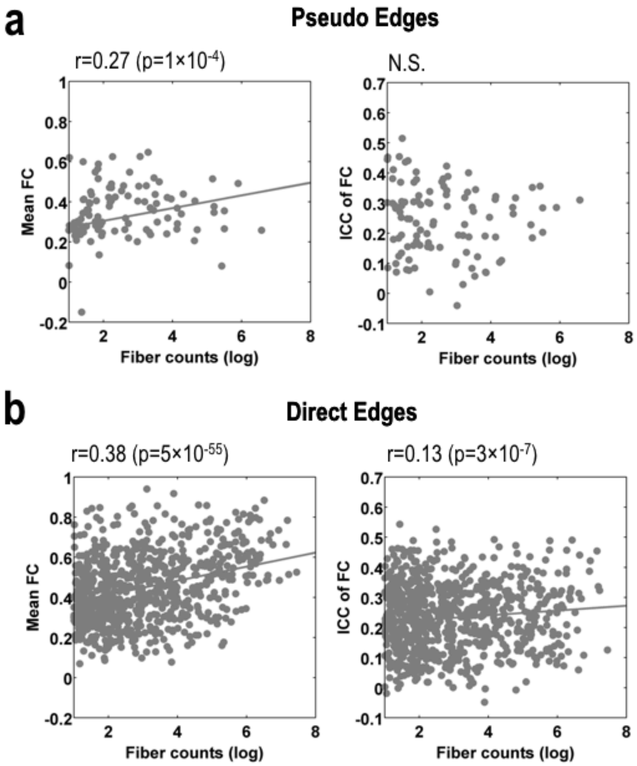

Figure S9. The relationships between structural connectivity and mean and stability of pseudo-FC and direct-FC after global signal regression. The relationships between the mean functional connectivity of FC and log-transformed fiber counts (left) and between the stability (ICC) of FC and log-transformed fiber counts (right) for pseudo edges (a) and direct edges (b).

Regions	Abbreviation	Regions	Abbreviation
Precental gyrus	PrCG	Lingual gyrus	LING
Superior frontal gyrus (dorsolateral part)	SFGdor	Superior occipital gyrus	SOG
Orbitofrontal gyrus (superior part)	OFGsup	Middle occipital gyrus	MOG
Middle frontal gyrus	MFG	Inferior occipital gyrus	IOG
Orbitofrontal gyrus (middle part)	OFGmid	Fusiform gyrus	FFG
Inferior frontal gyrus (opercular part)	IFGop	Postcentral gyrus	PoCG
Inferior frontal gyrus (triangular part)	IFGtr	Superior parietal gyrus	SPG
Orbitofrontal gyrus (inferior part)	OFGinf	Inferior parietal lobule	IPL
Rolandic operculum	ROL	Supramarginal gyrus	SMG
Supplementary motor area	SMA	Angular gyrus	ANG
Olfactory cortex	OLF	Precuneus	PRCU
Superior frontal gyrus (medial part)	SFGmed	Paracentral lobule	PCL
Orbitofrontal gyrus (medial part)	OFGmed	Caudate	CAU
Rectus	REC	Putamen	PUT
Insula	INS	Pallidum	PAL
Anterior cingulate cortex	ACC	Thalamus	THL
Middle cingulate cortex	MCC	Heschl gyrus	HES
Posterior cingulate cortex	PCC	Superior temporal gyrus	STG
Hippocampus	HP	Temporal pole (superior part)	TPsup
Parahippocampal gyrus	PHG	Middle temporal gyrus	MTG
Amygdala	AMYG	Temporal pole (middle part)	TPmid
Calcarine	CAL	Inferior temporal gyrus	ITG
Cuneus	CUN		

Table S1. Regional abbreviations of brain regions in AAL atlas.
